# Physical inactivity among corporate bank workers in Accra, Ghana: Implications for health promotion

**DOI:** 10.1371/journal.pone.0277994

**Published:** 2023-05-11

**Authors:** George Bediako Nketiah, Kwasi Odoi-Agyarko, Tom Akuetteh Ndanu, Frank Ekow Atta Hayford, Gordon Amoh, Henry Lawson

**Affiliations:** 1 Family Medicine/Polyclinic Department, Korle-Bu Teaching Hospital, Accra, Ghana; 2 RHI Medical Centre, Amanokrom-Akuapem, Ghana; 3 Department of Community and Preventive Dentistry, University of Ghana Dental School, Accra, Ghana; 4 Department of Dietetics, School of Biomedical and Allied Health Sciences, College of Health Sciences, University of Ghana, Accra, Ghana; 5 Department of Community Health, University of Ghana Medical School, Accra, Ghana; Universiti Malaya, MALAYSIA

## Abstract

**Introduction:**

Any type of activity that results in caloric expenditure has the potential to reduce the risk of cardiovascular diseases; nonetheless, most people, especially office workers, are physically inactive. This study sought to evaluate the extent of physical inactivity and its determinants among the staff of selected banks in Accra, Ghana.

**Methods:**

This was a cross-sectional study involving 219 banking staff randomly selected from five commercial banking institutions in Accra, Ghana. Demographic data was collected with a structured questionnaire. Physical inactivity was assessed using the Global Physical Activity Questionnaire. Study associations were determined using univariate analysis, and multivariate logistic regression models with adjusted odds ratio (AOR) and 95% confidence intervals (CI) estimated.

**Results:**

Two hundred and nineteen (219) participants were recruited, out of which 56.6% were males and 43.4% were females. The mean age (± SD) of the participants was 40.0±7.9 years. Physical inactivity was observed in 179 (81.7%) participants. The following were independently associated with physical inactivity: travel-related activities (AOR, 0.151; 95% CI, 0.059–0.384; p<0.001); working in the bank for 6–10 years (AOR, 4.617; 95% CI, 1.590–13.405; p = 0.005); and working in the bank for 11 years and above (AOR, 2.816; 95% CI, 1.076–7.368; p = 0.035).

**Conclusion:**

Physical inactivity was very high among bankers. Travel-related activities reduced physical inactivity whiles working at the bank for more than six years increased physical inactivity. Thus, promoting regular physical activity, frequent monitoring, and implementation of other appropriate healthy lifestyle intervention strategies are vital to reduce risk of early onset disease conditions associated with physical inactivity in this population.

## Introduction

Globally, the levels of physical inactivity continue to rise due to the development of new technologies which help humans perform certain activities with greater ease [1]. Some other reasons attributed to the high levels of physical inactivity include lack of time, financial costs, entrenched attitudes and behaviours, restrictions in the physical environment, low socioeconomic status and lack of knowledge [2]. Physical inactivity increases with age, is higher in adult women than men, and is more prevalent in high-income countries compared to low-income countries [3].

Office workers are even prone to higher levels of physical inactivity because they spend a greater part of their wakeful time (an average of 9.7 hours) in their office seats [4]. This lifestyle affords them little time to exercise and makes them sedentary. Sedentary behaviour encompasses activities like reading, driving, and watching television that are done while seated or reclined [5]. Physical inactivity and sedentary behaviours have been found in studies to have similar health hazards [5]. A person can be physically inactive but not necessarily sedentary. Conversely, one can be physically active but engage in high levels of sedentary behaviour.

To be physically active, the World Health Organization (WHO) recommends at least 150 minutes a week of moderate-intensity aerobic physical activity or 75 minutes a week of vigorous-intensity physical activity in adults [6] or an equivalent combination achieving at least 600 metabolic equivalent of task (MET). Another method of measuring physical activity at different levels of effort is MET and it is used to estimate the amount of oxygen used by the body during physical activity [7]. Physical activity and exercise have undebatable health benefits; virtually everybody can gain from being more physically active [8].

While people may engage in different domains of activities to meet their recommended activity levels, more than a quarter (28%) of the adult global population are physically inactive [1], which is slightly higher than the physical inactivity levels recorded among the general Ghanaian population (21.7%) [9]. Among the staff of some financial institutions in Ghana, about 80% were found to be physically inactive [10]. Physical inactivity is a primary but modifiable risk factor for non-communicable diseases, particularly cardiovascular diseases (CVDs) [11–13]. Physical inactivity remains one of the leading risk factors for worldwide mortality and accounts for over two-thirds (2.6 million) of the mortality in low- and middle-income countries [14]. Physical inactivity which is higher in office workers has also been found to be associated with higher incidences of CVDs and CVD risk factors, and reduced quality of life among these workers [15, 16]. Although some studies have looked at physical inactivity levels in the sub-region, only a few studies have looked at office workers, especially the banking population. In Ghana, where most office workers spend an average of 8 hours a day at work [17], bank workers report spending up to 12 hours a day at their workplaces [18]. This makes them vulnerable to higher levels of inactivity. To the best of our knowledge, no study has explored the domains of activities practiced among this working population in Ghana or determined factors associated with physical inactivity. The findings from this work will provide preliminary data that may help re-strategize approaches to health promotion among office workers with the ultimate goal of reducing physical inactivity and CVDs.

The study aimed to assess physical inactivity among bankers in Accra, Ghana. The specific objectives of the study were to: estimate physical inactivity; demonstrate the different domains of activity practiced; determine sedentary behaviour and evaluate factors that are associated with physical inactivity among these bankers.

## Methods

### Study design, population, and period

This was a cross-sectional study conducted between September 2018 and February 2019. The study was conducted at the head offices of selected commercial banking institutions in the Accra Metropolis of Ghana. The head office is the centre of operations of all commercial banks and harbours all departments of the institution. Bankers constitute a category of office workers with sedentary behaviour that increase the risk of CVDs [19]. Bankers include Tellers, customer service personnel, audit and inspection officers, treasury personnel, human resource personnel, marketing personnel, accounting officers, risk and compliance officers, credit analysts, executive managers, and information technology personnel.

### Sampling, inclusion, and exclusion criteria

At the time of study, there were thirty-four (34) registered commercial banks in Ghana and thirty (30) of them had head offices situated in the Accra metropolis [20]. From an alphabetically sorted and numbered list of all commercial banking institutions in Accra, five were selected as study sites using the random number generator function in Statistical Package for Social Sciences (SPSS), version 25. Using the same method, forty-five participants were randomly recruited from each selected bank. The following banking staff were excluded from the study: Pregnant women, those who joined the banking industry for the first time within 6 months before the study and those who were on routine annual leave during the study period.

### Data collection, measurements, and quality control

Data collection was done at the premises of the selected banks. Participants completed a structured questionnaire which included sociodemographic questions (sex, age, etc.) and the Global Physical Activity Questionnaire(GPAQ) to measure physical activity [21]. This consisted of open- and close-ended questions. The first part of the questionnaire focused on demographic information of the participants, which included gender, age, highest education level and the number of years working in the bank.

The second part measured the physical activity levels of participants across three domains: work‐related (home /office) activities, travel‐related activities, and recreational‐related activities undertaken for at least 10-minutes continuously. Work-related activities included heavy cleaning, gardening, carrying heavy loads, construction work, digging etc. Travel-related activities included walking or using bicycles to get to and from places, while recreational-related activities included brisk walking, jogging, aerobics, cycling, soccer, lawn tennis, volleyball, etc. These were further categorized into moderate- and vigorous-intensity activities. Examples of vigorous-intensity physical activity included soccer game, fast cycling, running/jogging, hiking uphill, carrying heavy loads, heavy gardening, and aerobic dancing. Moderate-intensity physical activity also included heavy cleaning or washing, carrying light loads, general gardening, light cycling, mowing lawns, brisk walking, and tennis doubles. For the calculation of participants’ overall energy expenditure, the time in minutes for each intensity was multiplied by the MET values for corresponding activities as specified by WHO: where moderate-intensity activity is equivalent to a MET value of 4.0 and vigorous-intensity activity is equivalent to a MET value of 8.0 [7]. The total METs expended in moderate and vigorous physical activity was used to sort participants into two physical activity categories as recommended by the WHO [6]. Example: a participant who engaged in 30 minutes of moderate-intensity work-related activity, 60 minutes of moderate-intensity recreational-related activity and 30 minutes of vigorous-intensity recreational-related activity a week, will have a total of 600 METs [(30 x 4) METs + (60 x 4) METs + (30 x 8) METs)] a week. Sedentary behaviour was assessed by the number of hours participants spend sitting or reclining on a typical working day. Sedentary behaviour was assessed as a categorical variable as high (≥8 hours per day) and normal (< 8 hours per day) [22].

### Statistical analysis

Study data was analyzed using Statistical Package for Social Sciences (SPSS) version 25. Study outcomes were determined using uni- and bi-variate analysis, and multivariate logistic regression models. Comparisons between categorical data were done with Chi-square or Fisher’s exact test where appropriate. Continuous data (skewed) were compared using the Kruskal-Wallis test and the post hoc analysis was done using Dunn’s multiple comparison test. The level of statistical significance was set at p < 0.05. Socio-demographic characteristics and the domains of activity that showed significant bivariate associations (P-value <0.05) with physical inactivity were further analyzed using multivariate logistic regression to identify independent risk factors associated with physical inactivity.

### Ethical considerations

This study received ethical clearance from the Ghana Health Service Ethics Review Committee (GHS-ERC011/05/15). Study was adequately explained to participants and written consent sought before they were recruited. Permission was also sought from the management of all the banks selected for the study.

## Results

Overall, 225 professional bankers (45 each from 5 selected banks) were invited to participate in the study. Of these, 219 bankers responded and were included in the study, representing a response rate of 97.3% [95% CI: 94.3–98.7]. Of the 219 participants, 56.6% were males and 43.4% were females. The age of participants ranged from 26-60years with a mean age (±SD) of 40.0±7.9 years. Participants aged between 20-39years were 119 (54.3%) and those aged between 40–60 years were 100 (45.7%). All the participants had worked in a bank for 2-37years. Forty-five (20.5%) of the participants had worked in a bank for 1–5 years; 68 (31.1%) had worked for 6–10 years; and 106 (48.4%) had worked for more than 10years. All participants had spent between 15years and 30years in formal education after preschool.

**Fig 1:**
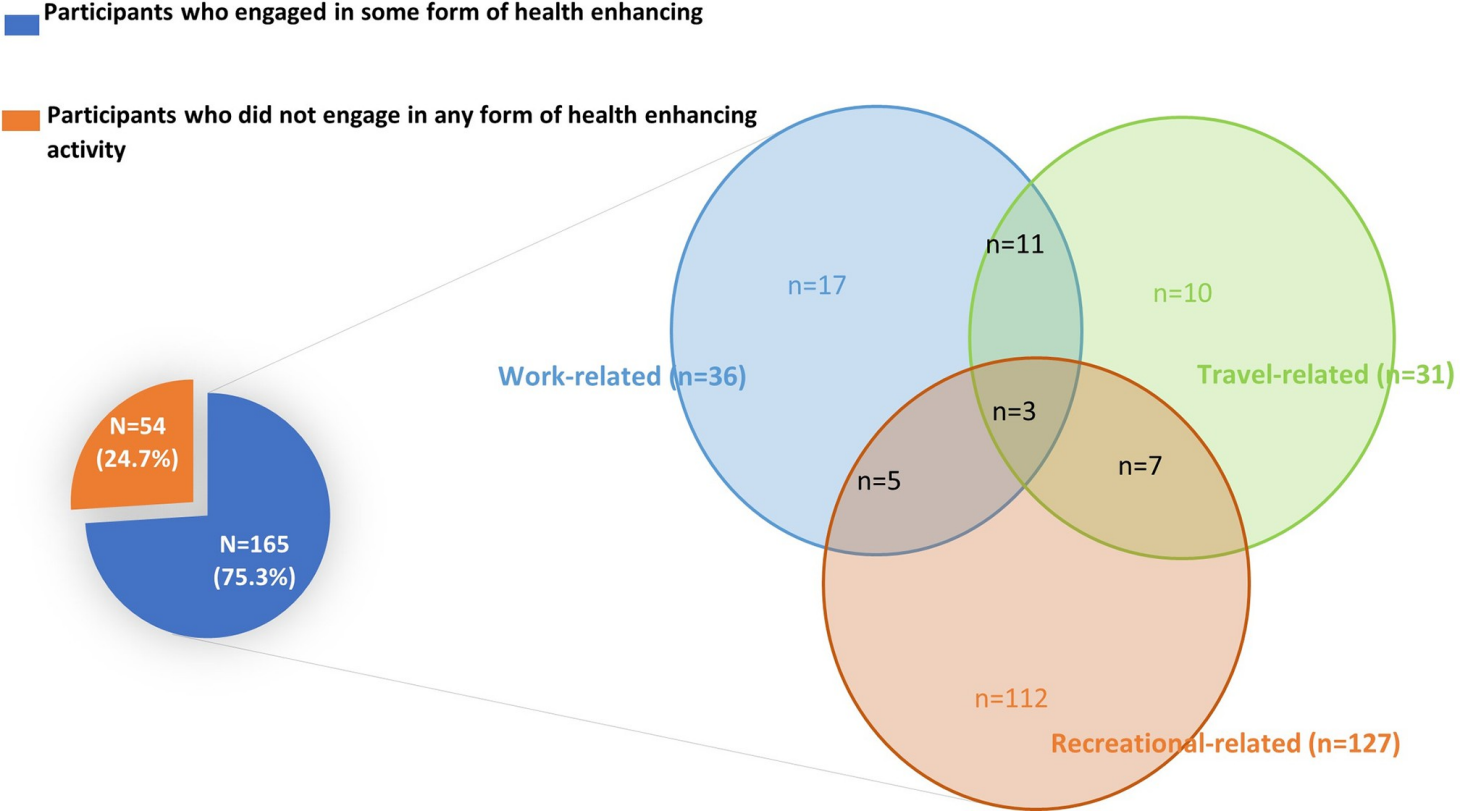
Domains of health enhancing activities. The Venn diagram shows the various domain of activities practiced either alone or in combination as illustrated by the intersection of the sets.

**Fig 2:**
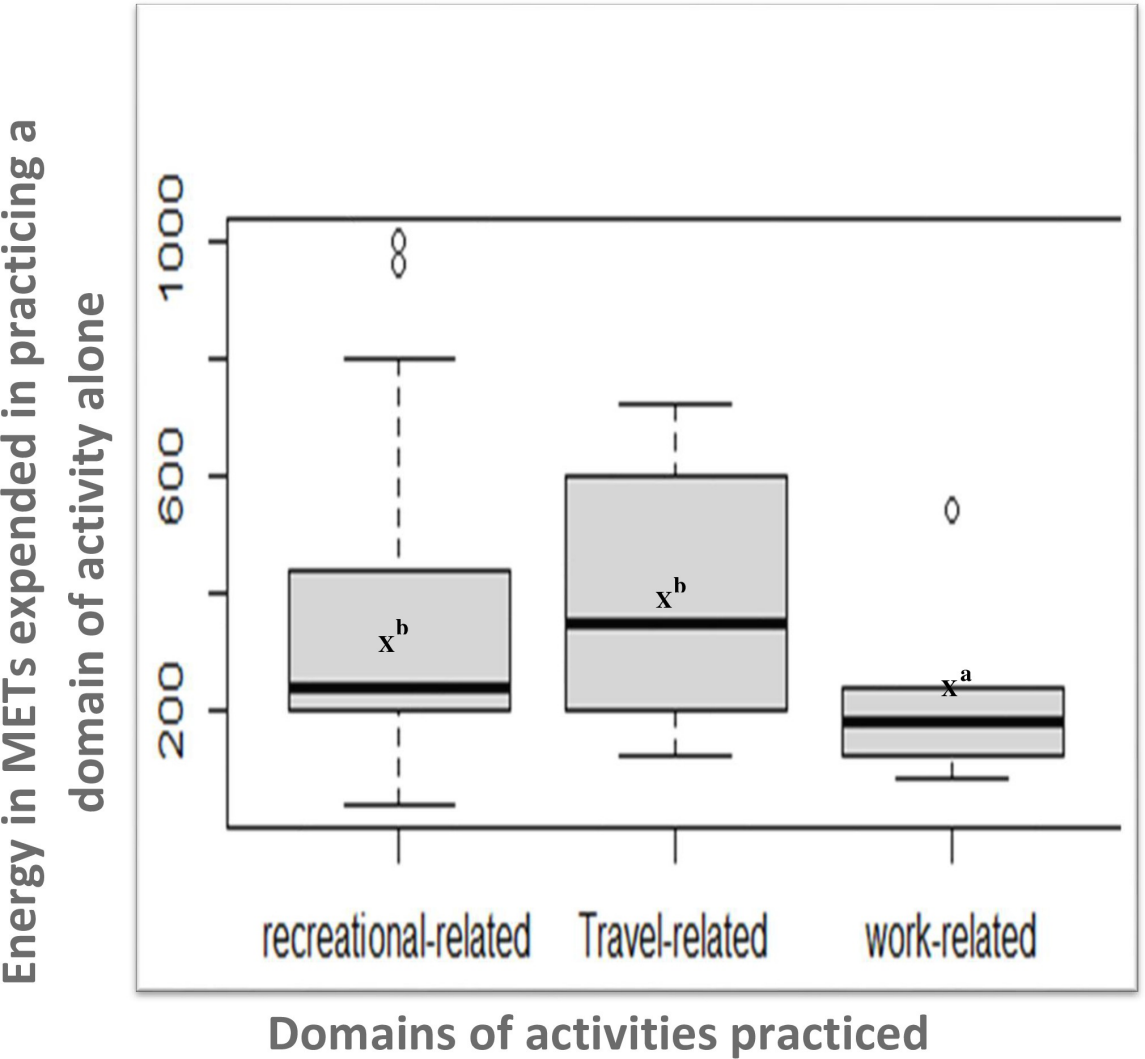
Domains of activities practiced and energy in METs expended in practicing a domain of activity alone. The box plot shows the interquartile range, horizontal line in the box shows the median of the distribution, X denotes mean of the distribution, lower whisker shows 25th percentile or lower quartile, upper whisker shows 75th percentile or upper quartile. NB. Superscripts compare the mean METs per week expended per domain of activities practiced alone using Dunn’s multiple comparison test, where b>a (P<0.05).

The prevalence of physical inactivity among the study participants was 81.7%. Altogether, 165 (75.3%) of the study participants indulged in some form of physical activity, either work-related, travel-related, recreational-related or a combination of these (Fig 1). Out of the total participants, 10 participants engaged in travel-related activities alone, 17 in only work-related activities and 112 in only recreational-related activities. The mean (±SD) METs per week expended by banking staff who engaged in only travel-related activities (378.0±205.6), or only recreational-related activities (336.6±208.9) was significantly higher than what was expended by banking staff who engaged in only work-related activities (196.5±106.4); *p* < 0.05 as shown in Fig 2 and Table 1. Overall, the mean (±SD) amount of time spent by banking staff in a sedentary behaviour was 8.66±2.10 hours/day. About 159 (69%) of the banking staff indulged in high sedentary behaviour (Table 2).

**Table 1:** Comparison between participants who engaged in only travel-related activities, only work-related activities, and only recreational-related activities.

	Domains of activities	Domains of activities	z value	p value
**Dunn’s multiple comparison test**	Recreational-related	Travel-related	-0.7006	0.7253
	Recreational-related	Work-related	3.0885	0.0030
	travel-related	Work-related	2.5974	0.0141

**Table 2:** Sedentary behaviour among banking staff.

	Normal sedentary behaviour	High sedentary behaviour	Overall
**Number of bankers**	68 (31.1%)	151 (68.9%)	219
**Mean time in hours per day (± standard deviation)**	6.54 ± 0.63	9.64 ± 1.74	8.66 ± 2.10
**Range**	5.0–7.0	8.0–14.0	3.0–14.0
**Interquartile range (25%, 75%)**	6.0, 7.0	8.0, 11.0	7.0, 10.0

NB. Sedentary behaviour was categorized as high (≥8 hours per day) and normal (< 8 hours per day)

In the univariate model (Table 3), the prevalence of physical inactivity was significantly higher in participants who had worked in the bank for 6–10 years or greater than 10 years, compared to those who had worked in the bank for 1-5years. The prevalence of physical inactivity did not differ significantly between participants who were 40–60 years compared to those between 20–39 years. The prevalence of physical inactivity did not also differ significantly between males and females nor among the various banks. Compared to participants who did not engage in travel-related activities, those who engaged in travel related activities were less likely to be physically inactive. Likewise, compared to participants who did not engage in work-related activities, those who engaged in work-related activities were less likely to be physically inactive. There was no significant difference in the prevalence of physical inactivity among participants who engaged in recreational-related activities compared to those who did not engage in them. In the logistic regression analysis using physical inactivity as the dependent outcome, the following variables were significant independent predictors of physical inactivity: working at the bank for 6–10 years (AOR, 4.617; 95% CI, 1.590–13.405; *p* = 0.005) or greater than 10 years (AOR, 2.816; 95% CI, 1.076–7.368; *p* = 0.035) and travel-related activities (AOR, 0.151; 95% CI, 0.059–0.384; *p*<0.001). All these were adjusted for in addition to work related activities (Table 3).

**Table 3:** Factors associated with physical inactivity.

Category		Physically inactive	Univariate model	Multivariate model	
		Frequency (%)	Crude OR (95% CI)	Adjusted OR (95%CI)	P-value
**Bank group**					
	Bank 1(45)	40 (88.9)	Reference		
	Bank 2(45)	36 (80.0)	0.500 (0.153–1.631)		
	Bank 3(44)	36 (81.8)	0.563 (0.169–1.876)		
	Bank 4(44)	34 (77.3)	0.425 (0.132–1.365)		
	Bank 5(41)	33 (80.5)	0.516 (0.154–1.727)		
**Gender**					
	Male (124)	105 (84.7)	Reference		
	Female (95)	74 (77.9)	1.568 (0.788–3.121)		
**Age**					
	20–39 years (119)	92 (77.3)	Reference		
	40–60 years (100)	87 (87.0)	1.964 (0.953–4.050)		
**years worked at the bank**					
	1- 5years (45)	25 (55.6)	Reference	Reference	
	6-10years (68)	61 (89.7)	6.971 (2.620–18.548) *	4.617(1.590–13.405)	**0.005**
	11years and above (106)	93 (87.7)	5.723 (2.505–13.075) *	2.816(1.076–7.368)	**0.035**
**Work-related activities**					
	No activities (183)	157 (85.8)	Reference	Reference	
	Perform activity (36)	22 (61.1)	0.260 (0,118–0.572) *	0.606(0.225–1.631)	0.322
**Travel-related activities**					
	No activities (188)	166 (88.3)	Reference	Reference	
	Perform activity (31)	13 (41.9)	0.096 (0.041–0.222) *	0.151(0.059–0.384)	**<0.001**
**Recreational-related activities**					
	No activities (92)	80 (87.0)	Reference		
	Perform activity (127)	78.0)	(0,254–1.109)		

OR; Odds Ratio; CI, Confidence Interval

*statistically significant at (α<0.05)

NB. Only variables with significant associations in the univariate model were put in the multivariate model

## Discussion

Physical inactivity continues to saddle society with a hidden and growing cost of healthcare and loss of productivity [1]. This study assessed physical inactivity among banking staff in Accra. The age range of the study population (26-60years) was characteristic of the typical working-age group in Ghana [23]. All the participants had spent at least 15 years of formal education after preschool which presupposes that most participants had received tertiary education.

The study reported a very high prevalence of physical inactivity (81.7%) among banking staff compared to the prevalence of 21.7% estimated for the general Ghanaian population from the study by Afrifa-Anane *et al*. [9]. This contrast may be due to the stressful nature of the banking profession coupled with their long working hours [24]. This lifestyle denies bankers adequate time for workouts. In a recent report on the barriers to the uptake of healthy behaviours, Kelly *et al* [2] described inadequate time for exercise as a major reason for recent high levels of physical inactivity. The high level of physical inactivity observed in this study is worrying, because of its strong association with high cardiovascular mortality [25, 26] however, it agrees with the 83.3% prevalence reported among workers in some financial institutions in Accra in 2015 [10]. In a similar study among bankers in Nigeria, Aderibigbe and colleagues observed a 60% prevalence of physical inactivity which is lower than what was recorded in the current study [27]. It is important to note that the prevalence of physical inactivity in this study was comparable across the five banks studied. This observation is in keeping with data from global reviews and across Ghana in particular which suggest that working conditions that predispose staff to sedentary behaviour and physical inactivity are similar in most banks [24, 28]. It’s noteworthy that, 57 of the 179 physically inactive banking staff did not indulge in any form of activity lasting at least 10minutes. The remaining 122 bankers practiced some form of activity, nevertheless, these activities were not enough to meet the recommended level to be considered physically active. This observation may be attributable to the inadequate time available to banking staff for exercise

Overall, a total of 165 study participants indulged in some form of activity lasting at least 10 minutes. The three domains of activities measured by the GPAQ were recreational-, work-, and travel-related activities. Recreational-related activities were the predominant (58%) domain of activity practiced by the bankers. This observation somewhat supports the finding by Nang *et al*. who reported that recreational-related activities are often the mainstay of persons within the higher socioeconomic bracket [29]. Beenackers *et al*. also reported in their systematic review that people of a high socioeconomic class are more likely to engage in recreational-related activities than people of low socioeconomic class because of resource availability and access to recreational facilities [30]. This may be the case of banking staff as most of them have access to recreational facilities. Fewer participants indulged in significant work- or travel-related activities. Most bankers belong to the high socioeconomic group of workers and are likely to have personal cars or house-helps which may reduce their travel-related and/or work-related activities at home. The sedentary nature of their work does not support work-related activity at the workplace. This supports the hypothesis of a changing social pattern of physical activity, with a decline in occupational physical activity, especially among the workers in the formal sector [31]. The use of personal cars as modes of transport, house-helps, and workplace technologies have been found in the study by Owen *et al*. to reduce work- and travel-related activities, especially among persons of high socioeconomic status [32]. Although WHO recommends physical activity across all domains to achieve the recommended activity levels, only 26 of the participants in this study indulged in two (2) or more domains of activity. Nang *et al*. also demonstrated in their study that engaging in multiple domains of activity increases the chances of achieving higher physical activity [29]. This may explain why the prevalence of inactivity was high among the banking staff although majority engaged in recreational-related activities.

In this study, banking staff who engaged in only travel-related or recreational-related activities had significantly higher total energy expenditure (METs) compared to banking staff who engaged only in work-related activities. If bankers are given the option to choose one of the domains of activities as their preferred physical activity, choosing travel- or recreational-related activities may help them expend higher levels of energy.

Nearly 70% of the participants indulged in highly sedentary behaviour. This prevalence is considerably higher than that reported in the general population (8.3%) for low and middle-income countries [33]. Over the past decade, there is increasing literature to demonstrate a strong morbidity risk associated with a sedentary behaviour, with some studies putting the sedentary behaviour at the same level as smoking [34–36]. The average estimated time spent in sedentary behaviour among the participants was 8.66 hours per day. This result, coupled with the high level of physical inactivity among participants, it is safe to suggest that working in banks predisposes individuals to very high health risks.

In this study, travel-related activities and working in the bank for more than 6 years were independently associated with physical inactivity. Whiles travel-related activities reduced the risk of being inactive by about 85%, working at the bank between 6 and 10 years increased the risk of being inactive by five-fold and working at the bank for more than 10 years increased the risk of inactivity by three-fold. The findings are consistent with a study by Knell *et al* [37], who concluded that travel-related activities were associated with high predictability (AOR, 7.3: 95% CI, 2.6–20.1) of meeting the physical activity recommendation. Salquist *et al*. [38] also concluded in their study that, substantial physical activity can be accumulated through travel-related activities. Travel-related activities generally involve longer durations of time and can help achieve the recommended levels of physical activity. Travel-related activities should, therefore, be encouraged to promote physical activity. The authors were unable to provide an explanation for why there was a decrease in the likelihood of being inactive among employees who had been with the bank for more than ten years. Further studies are suggested to answer this question.

Some workers have identified other independent predictor variables for physical inactivity. In a study by Mengesha *et al*. in Ethiopia, being a female adult and having office employment were significantly associated with physical inactivity [39]. Another study by Gichu *et al*. in Kenya found females to be more inactive than males, and adults aged 30–49 years were more inactive than their 50–69 year old peers [40]. This study however did not find female gender or age to be an independent predictor of physical inactivity.

Based on the findings of this study, we recommended that banking staff are encouraged to add travel-related activities to their domain of activities practiced because the results show that bankers who practiced travel-related activities achieved higher energy in MET per week. Travel-related activities independently decreased the likelihood of being physically inactive among the banking participants. Travel-related activities could, therefore, help bankers meet their recommended physical activity levels for health. Additionally, comprehensive workplace health promotion is recommended for all banking institutions to help improve healthy behaviours, particularly physical activity. Since bankers spend most of their wakeful time at the workplace, worksite health programs that promote physical activities will go a long way to help.

The study had some limitations that are worth noting. Firstly, data collected on participant’s activity levels were based on self-report and thus liable to recall bias. Secondly, the study sample comprised banking staff recruited from a regional district in Ghana. The study findings should therefore be interpreted with caution and may not be extrapolated to the generalized Ghanaian population. Nonetheless, the study highlights an important problem among office workers in Ghana. Thirdly, the study did not discriminate participants by departments of the banking institutions. The authors did not have that data for analysis. Future studies may be needed to investigate possible differences in physical inactivity among staff from different departments. This may help in redesigning specific health promotion programs for each department.

## Conclusion

The study reported high levels of physical inactivity among bankers, with majority indulged in high sedentary behaviour. The predominant domain of activity practiced by the participants was recreational-related activities. Those who engaged in either travel-related or recreational-related activities alone significantly expended higher energy in METs per week compared to those who practiced work-related activities alone. Engaging in travel-related activities decreases the likelihood of being inactive. Then again, working at the bank for more than six years increases the likelihood of being inactive. Hence there is the need for a concerted effort to encourage regular physical activity both outside and around the working environment and to implement other healthy lifestyle practices, as well as the continued monitoring of these behaviours, to prevent early onset of non-communicable diseases among this population.

## Supporting information

S1 File Study’s minimal underlying data set. (XLSX)

S2 File Study questionnaire. (PDF)
