# Supplementary material for: Physical inactivity among corporate bank workers in Accra, Ghana: Implications for health promotion
Source: PLoS One. 2023 May 11;18(5):e0277994. doi: 10.1371/journal.pone.0277994 (PMC10174574; doi:10.1371/journal.pone.0277994)
Supplement: S2 File — (PDF) [file pone.0277994.s002.pdf]

QUESTIONNAIRE

Topic: Physical inactivity among corporate bank workers in Accra, Ghana: Implications for health promotion

Survey Information		
**Consent, Interview Language and Name**	**Response**	**Code**
Consent has been read and obtained	Yes 1 No **If NO, END**	I 1
Interview Language *[Insert Language]*	English 1 [Add others] 2	I 2
Time of interview (24-hour clock)	Hrs.……...: min…………	I 3
Participants Identification Number	……………………………	I 4
Contact Phone Number where possible	+233……………………...	I 5
**Step 1 Demographic Information**		
**Demographic and work Information**		
Sex (Record Male / Female as observed)	Male 1 Female 2	C1
How old are you?	Years ………………...	C2
How long have you worked in the banking industry		C3
Past work history if any		C4
Have you suffered a cardiovascular event like stroke or coronary heart disease (heart attack) in the past?		C5
In total, how many years have you spent at school and in full-time study (excluding pre-school)?	Years ………………...	C6
**Step 2 - Measurement of physical activity**		
**Physical Activity**		
I will ask you about the time you spend doing different types of physical activity in a typical week.		
**Question**	**Response**	**Code**
**Work**		
Does your work (home/workplace) involve **vigorous-intensity** activity that causes large increases in breathing or heart rate like *[**carrying or lifting heavy loads, digging or construction work**]* for at least **10 minutes** continuously?	Yes 1<br>No 2 *If No, go to code P 4*	P1
In a typical week, on how many **days** do you do vigorous-intensity activities as part of your work?	Number of days ………………	P2
How much **time** do you spend doing vigorous-intensity activities at work on a typical day?	Hours: minutes: hrs.……Min…….	P3
Does your work (home/workplace) involve **moderate-intensity** activity, that causes small increases in breathing or heart rate such as ***carrying light loads or cleaning the house or gardening*** for at **least 10 minutes** continuously	Yes 1<br>No 2 *If No, go to code P 7*	P4

In a typical week, on how many **days** do you do moderate-intensity activities as part of your work?	Number of days ………………	P5
How much **time** do you spend doing moderate-intensity activities at work on a typical day?	Hours: minutes: hrs.…: min……	P6
**Travel to and from places**		
Now I would like to ask you about the usual way you travel to and from places. For example, to work, for shopping, to market, to place of worship		
Do you **walk or use a bicycle *(pedal cycle)*** for at least **10 minutes** continuously to get to and from places?	Yes 1<br>No 2, If No, go to code P 10	P7
In a typical week, on how many **days** do you walk or bicycle for at least 10 minutes continuously to get to and from places?	Number of days ………………	P8
How much **time** do you spend walking or bicycling for travel on a typical day?	Hours: minutes, hrs.…: min……	P9
**Recreational activities**		
Now I would like to ask you about sports, fitness and recreational activities (leisure),		
Do you do any **vigorous-intensity sports, fitness or recreational *(leisure)*** activities that cause large increases in breathing or heart rate like ***[running or football]*** for at least **10 minutes** continuously?	Yes 1<br>No2 *If No, go to code P 13*	P10
In a typical week, on how many **days** do you do vigorous-intensity sports, fitness or recreational *(leisure)* activities?	Number of days ………………...	P11
How much **time** do you spend doing vigorous-intensity sports, fitness or recreational activities on a typical day?	Hours: minutes, hrs.….: min…….	P12
Do you do any **moderate-intensity sports, fitness or recreational *(leisure)*** activities that cause a small increase in breathing or heart rate such as brisk walking, ***cycling, swimming, volleyball etc*** for at least **10 minutes** continuously?*)*	Yes *1*<br>No 2 *If No, go to code P16*	P13
In a typical week, on how many **days** do you do moderate-intensity sports, fitness or recreational *(leisure)* activities?	Number of days ………………	P14
How much **time** do you spend doing moderate-intensity sports, fitness or recreational *(leisure)* activities on a typical day?	Hours: minutes, hrs.…. mins……	P15
**Sedentary behaviour**		
The following question is about sitting or reclining at work, at home, getting to and from places, or with friends including time spent sitting at a desk, sitting with friends, traveling in car, bus, train, reading, playing cards or watching television, but do not include time spent sleeping.		
How much **time** do you usually spend **sitting or reclining** on a typical day?	Hours: minutes, hrs.……min…...	P16
